# Combined inhibition of MEK and nuclear ERK translocation has synergistic antitumor activity in melanoma cells

**DOI:** 10.1038/s41598-017-16558-0

**Published:** 2017-11-27

**Authors:** Rand Arafeh, Karen Flores, Alona Keren-Paz, Galia Maik-Rachline, Naomi Gutkind, Steven Rosenberg, Rony Seger, Yardena Samuels

**Affiliations:** 1 Weizmann Institute of Science, Rehovot, Israel; 2 National Cancer Institute, NIH, Bethesda, MD 20892 USA

## Abstract

Genetic alterations in *BRAF*, *NRAS* and *NF1* that activate the ERK cascade, account for over 80% of metastatic melanomas. However, ERK cascade inhibitors have been proven beneficial almost exclusively for *BRAF* mutant melanomas. One of the hallmarks of the ERK cascade is the nuclear translocation of ERK1/2, which is important mainly for the induction of proliferation. This translocation can be inhibited by the NTS-derived peptide (EPE) that blocks the ERK1/2-importin7 interaction, inhibits the nuclear translocation of ERK1/2, and arrests active ERK1/2 in the cytoplasm. In this study, we found that the EPE peptide significantly reduced the viability of not only *BRAF*, but also several *NRAS* and *NF1* mutant melanomas. Importantly, combination of the EPE peptide and trametinib showed synergy in reducing the viability of some *NRAS* mutant melanomas, an effect driven by the partial preservation of negative feedback loops. The same combination significantly reduced the viability of other melanoma cells, including those resistant to mono-treatment with EPE peptide and ERK cascade inhibitors. Our study indicates that targeting the nuclear translocation of ERK1/2, in combination with MEK inhibitors can be used for the treatment of different mutant melanomas.

## Introduction

Malignant melanoma is the most fatal type of skin cancer^1^. The incidence of melanoma continues to increase, and represents a significant health problem worldwide^2^. Over the past decade, comprehensive sequencing efforts that shed light into the melanoma genetic landscape have enabled the discovery of several novel driver genes^3–5^. Melanomas are divided into four different subgroups depending on their driving mutation’s status. The first group include *BRAF* (most often *BRAF* ^*V600E*^) mutant melanomas (~50%)^6^, the second group are *NRAS* ^*Q61L/R*^ mutant melanomas (15–20%), the third group are *NF1* mutant melanomas (15%), and the fourth group are triple wild-type melanomas (15–20%)^7^. The driving mutations of the first three subgroups are all known to hyperactivate the ERK cascade^3^, making it a favorable potential candidate for targeted therapy, considering ERK1/2 itself as the ‘best’ node for effective interruption of ERK signaling^8^.

The identification of these mutations motivated the development of targeted drugs^6,9^ against different tiers of the ERK cascade. Efforts to develop RAS inhibitors have mostly failed, with no targeted therapy against this protein so far^10,11^. However, inhibitors of BRAF, MEK1/2 and recently also ERK1/2, have been developed in the past year^12–14^. Although the initial response rate to Vemurafenib is more than 70%, with significant survival benefit, tumor resistance occurs within 2–18 months of treatment^15,16^. Although MEK mutations in melanoma occur rarely (~1%)^17^, its activity is elevated in almost all melanomas. Recent efforts have led to the development of the MEK inhibitor Trametinib^18^. In phase II clinical trials, trametinib treatment showed significant clinical benefit in *BRAF* melanoma patients who had not been previously treated with a BRAF inhibitor and minimal activity in sequential therapy in patients previously treated with BRAF inhibitors^19^. These trials initiated a new therapeutic strategy of combining RAF and MEK inhibitors. Indeed, the combination of dabrafenib and trametinib improved anti-tumor activity and survival in *BRAF* mutant melanoma patients^20^. Concurrently, immunotherapy has transitioned from cytokine-based treatment to antibody-mediated blockade of the cytotoxic T-lymphocyte-associated antigen-4 (CTLA-4) and the programmed cell-death protein 1 (PD-1) immune checkpoints^21–24^. These changes in the treatment landscape have dramatically improved patient outcomes, with the median overall survival of patients with advanced-stage melanoma increasing from approximately 9 months before 2011 to at least 2 years and probably longer for those with *BRAF* ^*V600*^ mutant disease^21,23^.

Although oncogenic mutations in *ERK1/2* are extremely rare^25^, its activity is elevated in about 85% of all cancers^6,26^. Therefore, it is still an attractive therapeutic target due to its central role in integrating signaling from various upstream components. A recently developed ERK1/2 inhibitor SCH772984^27^ showed benefits in reducing tumor growth in BRAF and MEK inhibitor- resistant models. Although inhibition of ERK1/2 mostly reduced cell growth of *BRAF* mutant melanomas, it also showed some partial reduction in *NRAS* and *KRAS* mutant cancer cell growth^27^. Several other ERK1/2 inhibitors are under development, but none of these compounds have been approved for clinical use. Moreover, these inhibitors were proven beneficial almost only in *BRAF* mutant melanomas^28,29^, and thus a considerable number of melanoma patients remain without a targetable mutation. Moreover, in patients that do respond to treatment, the heterogeneous nature of melanoma tumors leads to the rapid emergence of resistance^30–35^, due to escape mechanisms from the inhibitor’s blockade^36^, allowing cancer progression. Multiple mechanisms of resistance of *BRAF* mutant melanomas have been described, which can be classified as intrinsic^37^ or acquired^38,39^. These two types of drug resistance have been shown to result in either reactivation of the ERK1/2 signaling, failure to effectively deactivate ERK1/2, or activate alternative signaling pathways that overcome the inhibition of ERK1/2.

It was previously shown that the nuclear activity of ERK is mainly associated with cell proliferation^40^, whereas ERK negative feedback targets are mostly cytosolic^41^. Therefore, inhibition of nuclear ERK translocation, which reduces nuclear phosphorylaton without affecting much negative feedback loops, should result in inhibition of tumor growth with less or delayed resistance. In a previous study, some of us showed that stimulated nuclear translocation of ERK1/2, which is one of the hallmarks of the cascade, is mediated by phosphorylation of ERK’s Nuclear Translocation Signal (NTS) that consequently induces binding with importin7 that escorts active ERK1/2 into the nucleus^42–44^. It was later shown that by using a myristoylated NTS-derived phosphomimetic peptide (EPE peptide), the interaction of Importin7 with ERK1/2, and consequently the nuclear translocation of the latter, are inhibited. This inhibition then induces apoptosis of *BRAF* mutant melanoma cells, inhibits the proliferation/survival of many other cancer cells, including BRAF and MEK resistant melanoma cells, but has no effect on the viability of several immortalized cells^45^. In this study, we found that the EPE peptide also reduces the viability of *NRAS* and some *NF1* mutant melanomas as well, and combination of EPE peptide and MEK inhibitor trametinib showed synergy in reducing the growth of other melanomas, including those resistant to each drug alone. We also found that this synergistic effect of the drug combination is partially driven by the preservation of ERK1/2-mediated negative feedback loops. These findings have clinical implications as it may lead to development of a combined therapy of inhibitors of MEK and ERK1/2 nuclear translocation for a several types of melanoma patients.

## Results

### Effect of inhibition of nuclear ERK1/2 translocation by the EPE peptide on the viability of metastatic melanoma cells

**Figure 1:**
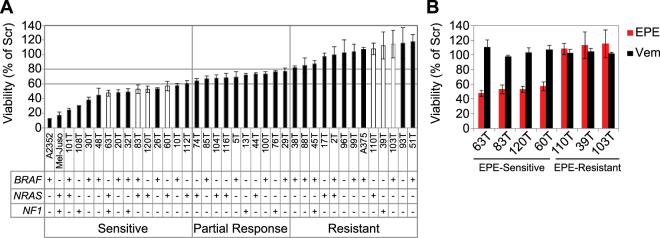
The EPE peptide reduces the viability of some *BRAF*, *NRAS* and *NF1* mutant melanoma cell lines. (**A**) Effect of the EPE peptide on proliferation of metastatic melanoma cells. Thirty-eight melanoma cell lines derived from metastatic tumor resections were treated with either EPE or scrambled (Scr) peptides (10 µM). A subset of *BRAF*, *NRAS* and *NF1* mutant melanomas were sensitive to the EPE peptide. Bars in white correspond to cell lines selected for further study. (**B**) Effect of BRAF inhibitor vemurafenib on selected melanomas. Four EPE-sensitive *NRAS* melanomas (63T, 83T, 120T and 60T), and three EPE-resistant melanomas (110T, 39T, 103T) were treated either with vemurafnib (1 µM, Vem), EPE or Scr peptides (10 µM). All melanoma cells selected were resistant to BRAF inhibition. Cell viability was measured by CellTiter-Glo reagent after 96 h of treatment. Bars represent percentage of growth respect to Scr peptide control ± S.E. of 3 independent experiments.

Previously, we have found that the EPE peptide inhibits the proliferation of several cancer cell types, and induces apoptosis in *BRAF* melanomas, while other types of melanoma were only partially affected^45^. In this current study, we extended the screen to include melanoma cells with a variety of mutational backgrounds, in order to assess the effect of the EPE peptide on the viability of different melanoma cells. We selected 38 melanoma cell lines, corresponding to the three major groups of mutations in melanoma: *BRAF* mutant, *NRAS* mutant, and *NF1* mutant melanomas^7^ (Supplementary Table 1), and evaluated their sensitivity to the EPE peptide according to the percentage of viability of EPE-treated cells compared to Scrambled (Scr) peptide control: Sensitive (below 60%), partial response (60%-80%) and resistant (above 80%) (Fig. 1). Interestingly, we found that the EPE peptide was effective not only in reducing the viability of many *BRAF* mutant melanomas, but it also affected some cell lines bearing *NRAS* and/or *NF1* mutations as well. Thus, some *NRAS* and *BRAF* mutant melanomas were sensitive to the EPE peptide, while others were either partially sensitive or not sensitive at all. Some of the *NF1* melanoma cells tested were very sensitive to the drug, but others were either partially responsive or did not respond to the EPE peptide. Two triple WT melanomas cell lines (93T and 96T) were included in the screen but they did not respond to the EPE peptide. Unexpectedly, more *NRAS* melanomas, that are less sensitive to clinical RAF and MEK inhibitors^46^, were found in the sensitive group compared to other groups (Seven *NRAS* mutant melanoma cell lines were found to be sensitive, compared to four melanoma cell lines that were partially sensitive and three melanoma cell lines that were completely unresponsive and resistant to EPE peptide) (Fig. 1). These results indicate that the EPE peptide affects the pERK-addicted melanomas, but likely requires some additional aberrations to exert its effects. Since *NRAS* mutant melanomas lack an effective targeted therapy^10^, we decided to continue our studies with four *NRAS* melanoma cell lines that were sensitive to EPE peptide (63T, 83T, 120T and 60T). In addition, we selected three EPE-resistant cell lines with diverse mutational backgrounds (110T – *NRAS* mutant; 39T – *NF1* mutant; and 103T – *BRAF* mutant) to shed light on the mechanism of resistance to EPE treatment (Fig. 1A, white bars). All EPE-sensitive *NRAS* melanomas selected were resistant to the BRAF inhibitor vemurafenib, while the EPE peptide reduced their viability to 50%. Importantly, all three EPE-resistant melanomas selected were also resistant to BRAF inhibition (Fig. 1B), indicating a common ERK related mechanism of resistance.

### Effect of the EPE peptide on nuclear ERK1/2 translocation and activity

**Figure 2:**
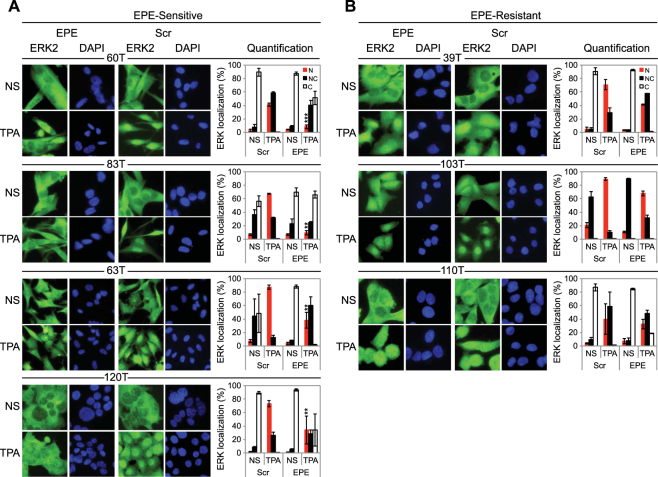
The EPE peptide blocks the nuclear translocation in “sensitive” *NRAS* melanomas. The same seven cell lines selected previously, were serum starved (14 h), pre-treated with EPE or Scr peptide (10 µM, 2 h), and stimulated with TPA (100 nM, 15 min) or left untreated (NS). Cells were then fixed and stained with αERK2 Abs and DAPI. **(A)** The EPE peptide significantly reduces the nuclear translocation of ERK1/2 in the sensitive cells. **(B)** The effect of EPE peptide on the nuclear translocation of ERK1/2 is modest in resistant cells compared to (A). Bars represent the average percentage of cells with mostly nuclear (N, red), nuclear and cytosolic (NC, black) or mostly cytosolic (C, white) staining. Error bars represent standard error of 2 or 3 independent experiments, ***p < 0.001, **p 0.001 to 0.01 (Student’s *t*-test). Quantification was done by counting at least three fields with > 150 cells.

In order to better understand the differences between EPE sensitive and resistant melanoma cells, we decided to examine whether there are differences in the ability of the EPE peptide to block the nuclear translocation of ERK1/2 in the different cells. In resting cells, ERK1/2 was predominantly localized in the cytosol (white bars) in six of the seven cell lines (Fig. 2). After 15 mins of TPA stimulation, all cell lines underwent ERK1/2 translocation to the nucleus. TPA is a known tumor promoter that mimics distinct intracellular events triggered by activated growth factors and it causes activation of the MAPK pathway^47^. In some cell lines ERK1/2 localization was mainly in the nucleus (red bars), or in some cases, presented an ‘all over’ distribution (equal nuclear and cytoplasmic localization) (black bars). Thus, the EPE peptide disrupted the normal stimuli-dependent nuclear translocation of ERK1/2 in all cases, but the extent of reduction on nuclear ERK1/2 localization (red bars) was much higher in sensitive cell lines (Fig. 2A and Supplementary Fig. 1C), compared to resistant cells (Fig. 2B). As for the triple WT melanoma cell lines, the EPE peptide was not efficient in disrupting the normal stimuli-dependent nuclear translocation of ERK1/2 (Supplementary Fig. 1A,B).

**Figure 3:**
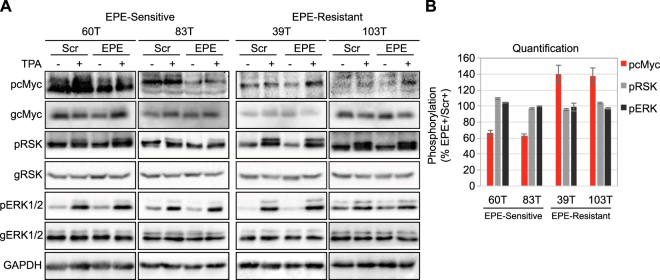
The EPE peptide reduces phosphorylation of nuclear targets in *NRAS* mutant melanomas. Two *NRAS* melanomas sensitive to EPE peptide (60T an 83T), and two EPE-resistant melanomas (39T and 103T), were serum starved (16 h), pretreated with EPE or Scr peptides (10 µM, 2 h), and stimulated with TPA (100 nM, 15 min) (+) or left untreated (−). Cell lysates where analyzed by WB using the indicated antibodies. **(A)** (*Left)* In the EPE-sensitive cells, the peptide reduced the phosphorylation of nuclear target c-Myc in stimulated and basal state, while not affecting cytosolic target RSK. *(Right)* In EPE-resistant cells, the peptide had no effect on the phosphorylation of RSK, and slightly increased levels of phospho-c-Myc. (**B**) Quantification of bands in (**A**). Bars represent average band density ratio of EPE stimulated samples (EPE+) compared to Scr stimulated samples (Scr+), ±S.E. of 2 or 3 independent experiments. Bands were quantified using ImageJ.

We next examined the effects of EPE peptide on ERK1/2 signaling, especially the effect on the cytoplasmic target of ERK1/2 RSK^48,49^ and on the nuclear target of ERK1/2 cMyc^50,51^. Similarly to previous results^45^, the EPE peptide did not inhibit the activity of ERK1/2 as seen by the preservation of phosphorylation of the ERK1/2’s activatory TEY motif and its substrate RSK in all cell lines examined (Fig. 3A,B). In two of the sensitive cell lines, the EPE peptide reduced the phosphorylation of the nuclear target of ERK1/2 cMyc in basal and stimulated state. However, in EPE resistant cells, the peptide slightly increased the phosphorylation levels of cMyc (by 40%) (Fig. 3B). These results may suggest that a small amount of the EPE peptide can enter the cells, but these amounts have a small effect on the signaling of resistant cells. Therefore, the mechanism of EPE peptide resistance is most likely not related to faster degradation or an impediment of the peptide to enter the cells. Given that the EPE peptide slightly affects the nuclear translocation of ERK1/2 and the levels of pcMyc in resistant cells, it is likely that the resistance is caused by the small amount of peptide, although other mechanisms may be involved as well.

### Synergistic effect of Trametinib and EPE peptide combination in reducing the viability of melanoma cells

Although the response rates to treatment with BRAF inhibitor vemurafenib can reach 60–80% in *BRAF* ^*V600E*^ melanoma patients^16^, only a few patients achieve single-agent complete response. This is mainly due to a relatively rapid development of resistance to ERK cascade inhibitors. In most cases, patients who had a positive initial response to single treatment with ERK cascade inhibitors, eventually relapse and develop resistance within months to a year by accumulating additional driver mutations in their tumors or by finding other escape routes that usually reactivate the ERK cascade^15,52^. Use of drug combinations has the potential to address these different resistance mechanisms. We therefore tested whether combining the MEK inhibitor trametinib and the ERK1/2 nuclear translocation inhibitor EPE peptide, could overcome the drug resistance (or lack of response) that metastatic melanoma cells showed when treated with each drug individually.

**Figure 4:**
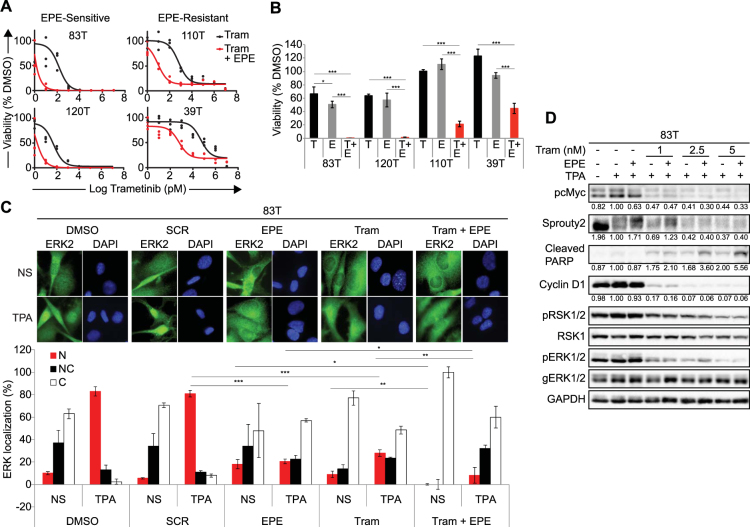
Synergistic effect of trametinib and EPE peptide combination in reducing the viability of melanoma cell lines. (**A**) Synergy between combined treatment of trametinib and EPE peptide in patient-derived melanoma cells. Dose response curves showing growth of melanoma cells treated with combination of trametinib and EPE peptide 10 µM (red), compared to trametinib treatment alone (black). Dots represent triplicates for every concentration point. (**B**) Effect of the combination of trametinib and EPE peptide on viability of metastatic melanoma cells lines. Cells were treated either with DMSO, trametinib alone (black), EPE peptide alone (10 µM, grey), or trametinib in combination with 10 µM EPE peptide (red) for 72 h. (trametinib concentrations per cell line: 83T and 120T − 10 pM; 110T – 0.1 nM; 39T − 1 nM). Viable cells were quantified using CellTiter-Glo reagent. Bars represent percentage of growth respect to DMSO ± S.E. of 2 independent experiments in triplicates, ***p < 0.001, *p < 0.01 (Student’s *t*-test) **(C)** Effect of EPE and trametinb combination on the nuclear translocation of ERK1/2. 83T cells were serum starved in the presence of EPE peptide, trametinib or EPE peptide and trametinib together for 24 hours. Cells were then stimulated or not with TPA (100 nM, 15 minutes). Cells were then fixed and stained with αERK2 Abs and DAPI. Bars represent the average percentage of cells with mostly nuclear (N, red), nuclear and cytosolic (NC, black) or mostly cytosolic (C, white) staining. Error bars represent standard error of 2 experiments, ***p < 0.001, **p 0.001 to 0.01 and *p < 0.01 (Student’s *t*-test). Quantification was done by counting at least three fields with > 150 cells. (**D**) Effect of the combination of trametinib and EPE peptide on ERK1/2 signaling. The combination increases ERK1/2 negative feedback loop mediated by Sprouty2 and apoptosis. *NRAS*-mutant melanoma cells 83T were serum starved in the presence of trametenib alone (1, 2.5 or 5 nM) or in combination with EPE peptide (10 µM) for 24 hours, and stimulated with TPA 100 nM for 15 min (+) or left untreated (−). Cell lysates where analyzed by WB using the indicated antibodies. Quantified levels are given under the blots. ImageJ was used for the quantification.

We observed a strong synergistic effect when combining trametinib and EPE peptide in all selected melanoma cell lines, including the EPE- and vemurafenib- resistant melanoma cells (*p* < 0.01*, p* < 0.001, Fig. 4A,B). The IC_50_ values of the combined treatment of trametinib and EPE peptide were close to 1 pM for the EPE-sensitive *NRAS* melanomas 83T and 120T, close to 10 pM for 110T and 1 nM for 39T (EPE-resistant melanomas). We observed a similar trend in the other cell lines (Supplementary Fig. 2). Therefore, the IC_50_ values for the combined treatment were two orders of magnitude lower, compared to trametinib alone, for all cell lines tested. Next, we tested the effect of the EPE peptide on nuclear ERK1/2 translocation in combination with trametinib compared to the single use of these drugs (Fig. 4C). In the presence of TPA, treatment with EPE alone or trametinib alone disrupted the normal stimuli-dependent nuclear translocation of ERK1/2, but this disruption was accentuated when combining EPE with trametinib. Interestingly, combining EPE and trametinib, in the absence of TPA stimulation, completely blocked the nuclear translocation and all cells were found to have a cytoplasmic ERK1/2 stain (Fig. 4C quantification).

In order to shed light on the mechanism that drives the synergistic effect, we examined the effect of the trametinib and EPE peptide combination on ERK cascade signaling in the EPE-sensitive 83T cells. Combination of both inhibitors resulted in a more profound decrease in the phosphorylation of ERK1/2 nuclear target cMyc, but had no significant difference on the phosphorylation of cytosolic target RSK and ERK-induced expression of Cyclin D1, compared to trametinib alone (Fig. 4D). Consistent with the synergy effect previously observed, combination of the inhibitors resulted in increased levels of cleaved PARP, a marker of apoptosis. Most importantly, combined treatment of EPE peptide and trametinib at very low concentrations, not only preserved, but also induced the phosphorylation of the transcription-dependent Sprouty2-mediated negative feedback loop of the ERK cascade as seen by the clear upshift of the Sprouty2 band. Sprouty2 is a known intracellular inhibitor of the ERK pathway^53–55^. This effect on pSprouty2 was not observed when trametinib was administered alone, or when it was combined with EPE peptide at higher combinations (Fig. 4D). These results suggest that the combinatorial use of trametinib and EPE peptide have a synergistic effect against different mutant melanoma cells.

## Discussion

The MAPK pathway is one of the most dysregulated pathways in melanoma but also in other cancers^56,57^. It is well known that ERK1/2 signaling plays a crucial role in the induction of proliferation, as well as cancer development and progression^58^. Therefore, it is not surprising that inhibitors of the ERK1/2 cascade (for example, vemurafenib and trametinib) serve as anticancer drugs^12,13^. However, despite high response rates, resistance to these drugs develops in the majority of patients and represents a major challenge in melanoma clinical practice^59^. Much of the shortcomings of the current inhibitors are probably mediated by inhibition of negative feedback loops. Therefore, as previously described^45^, some of us sought to prevent ERK1/2-dependent proliferation through the inhibition of its nuclear translocation. This way, cytosolic negative feedback loops would not be affected, and therefore the effects induced by their inhibition should be prevented. For this purpose, the Seger laboratory used the NTS-derived EPE peptide (NTS; Nuclear Translocation Signal), which competes for the interaction of Importin7 to ERK1/2. As a consequence, this peptide efficiently inhibits the nuclear translocation of ERK1/2, and was shown to induce apoptosis of *BRAF* mutant melanomas^45^.

Here, we show that the EPE peptide not only reduces the growth of many *BRAF* mutant melanomas, but also several *NRAS* and *NF1* mutant melanomas, insensitive to BRAF inhibition. Although it is clear that the EPE peptide exerts its effect on ERK-addicted cells, some of these cells were not affected by treatment with this peptide. We did not find a common denominator or correlation between the response of the cells to EPE and their different mutational backgrounds.

Further comparison between the selected melanoma cells, showed that the EPE peptide indeed reduced the stimulated nuclear translocation in all cell lines. However, the inhibitory effect of the EPE peptide on the EPE-resistant cells was less impressive compared to the EPE-sensitive cells. This reduced inhibitory effect may be due to two possible reasons. The first may be reduced amount of the EPE peptide in the cells, which could be due to faster clearance (e.g. by the multidrug resistant proteins (MDR) faster degradation, or masking by other proteins, phospholipids or vesicles). The second reason may be intrinsic mutations in the cell that circumvent the inhibitory effect by several methods including enhancing nuclear ERK1/2 translocation or affecting ERK1/2-related phosphatases, scaffolds and other signaling proteins. Because of the minor effects on cMyc, seen in the EPE-resistant cells, we believe that the most likely explanation is the first, namely the amount of peptide. However, more investigation is required to decipher the mechanism of resistance to the EPE peptide.

When analyzing the effect of the EPE peptide on ERK cascade signaling, we found that the EPE peptide significantly downregulates ERK1/2 nuclear targets in EPE-sensitive cells, on both basal and stimulated states. The effect on EPE resistant cells was much smaller and sometimes different, as in the case of cMyc (Fig. 3). The small elevation of pcMyc might suggest that the mechanism of resistance to the EPE peptide may be due to the ERK cascade itself. Therefore, we sought to examine whether combining the EPE peptide with the MEK inhibitor trametinib, would overcome the resistance to the EPE peptide. Indeed, combined inhibition of MEK and ERK nuclear translocation had a synergistic effect, reducing the viability of EPE sensitive *NRAS* melanomas and the EPE-resistant melanoma cells. In EPE-resistant cells, this effect was quite impressive since these cells were also much less sensitive to trametinib alone, but combination of trametinib and EPE peptide completely inhibited their cell growth at concentrations as low as 0.1–1 nM of trametinib. These results confirm that the EPE peptide enters the cells and is able to execute ERK1/2 nuclear translocation inhibitory effects. Moreover, combination of MEK inhibition at very low concentration and ERK1/2 nuclear translocation inhibition, resulted in a more profound decrease in the phosphorylation of ERK1/2’s nuclear target cMyc. The reason for the synergistic effect of the two compounds is not clear as yet. Our thoughts are that it is dependent on the maintenance of residual negative feedback loops after the treatment with the MEK inhibitor. In this sense, it is important to mention the phosphorylation of Sprouty2 and the increase of pSprouty2 in the combination treatment, which might be dependent on the hyperactivation of either RSK or other signaling molecules or pathways.

Our data then confirm the importance of the preservation of the negative feedback loops to overcome drug resistance. It also shows that combination of inhibitors of components of the ERK cascade together with inhibition of the nuclear translocation of ERK1/2, could be an effective treatment for different mutated metastatic melanomas.

## Methods

### Reagents

Tetradecanoyl phorbol acetate (TPA), poly-L-Lysine (PLL) and 4,6-diamino-2-phenylindole (DAPI) were purchased from Sigma-Aldrich (Rehovot, Israel). Albumin bovine serum (BSA) was purchased from MP biomedical (OH, USA). The BRAF inhibitor PLX4032 and MEK inhibitor Trametinib (GSK1120212) were purchased from SelleckChem (Huston, TX). CellTiter-Glo reagent was purchased from Promega (Madison, WI).

### Buffers

Sample buffer 2X: 2.5% SDS, 25% glycerol, 125 mM Tris Cl ph 6.8, 4% v/v β-mercaptoethanol, 0.01% bromophenol blue. TBST wash buffer: 200 mM Tris (pH 7.5), 1.5 M NaCl, 0.5% Tween 20.

### Antibodies

General RSK1 (C-21; 1:4000), pRSK1/2 (T359, S381; 1:2000), pcMyc (T58, S62; 1:1000) and GAPDH (FL-335; 1:1000) antibodies (Abs) were obtained from Santa Cruz Biotechnology (CA, USA). Anti-CyclinD1 (92G2, 1:1000) and PARP (46D11, 1:1000) Abs were obtained from Cell Signaling Technology (Beverly, MA, USA). Anti-pERK1/2 (1:20000) and gERK1/2 (1:20000) Abs were obtained from Sigma (Rehovot, Israel). Anti-Sprouty2 (aminoterminal, 1:1000) Ab was obtained from Abcam (Cambridge, UK). Secondary fluorescent Ab conjugates were obtained from Jackson ImmunoResearch (West Grove, PA). Secondary Abs conjugated to horseradish peroxidase (HRP) were obtained from Nichirei Biosciences (Japan).

### Peptides

The peptides used were: Scrambled (Scr), GNILSQELPHSGDLQIGL, and EPE: GQLNHILGILGEPEQEDL. Both peptides were N-terminal conjugated to myristic acid^60^ and C-terminal amidated, purchased from GenScript (HGK, China), purity >85% and kept as 100 mM in DMSO at −20 °C.

### Cells

Low-passage primary melanoma cells A2352 were from the Ella Institute, Sheba Medical Center, Israel. Established melanoma cell A375 was from ATCC.

### Tumor Tissue

A subset of cell lines used in the study (‘T’ cells) were derived from a panel of pathology-confirmed metastatic melanoma tumor resections collected from patients enrolled in institutional review board (IRB)- approved clinical trials at the Surgery Branch of the National Cancer Institute. These cell lines were established at the NCI with informed patient consent under on a clinical protocol (03-C-0277) approved by the institutional-review board (IRB) of the National Cancer Institute (NCI). All methods were performed in accordance with institutional guidelines and regulations (Weizmann Institute of Science, Bioethics Committee). Pathology-confirmed melanoma cell lines were derived from mechanically or enzymatically dispersed tumor cells, which were then cultured in RPMI-1640 supplemented with 10% FBS at 37 °C in 5% CO2 for 5–15 passages. Cell line genotypes are given in Supplementary Table 1. All cell lines have tested negative for mycoplasma.

### PCR sequencing and mutational analysis

PCR and sequencing of *BRAF, NRAS* and *NF1* were carried out as previously described^61,62^.

### Fluorescence microscopy

Cells were seeded on coverslips coated with 0.001% w/v poly-L-Lysine (PLL) at 60% confluency. After treatments, cells were fixed in 4% paraformaldehyde/PBS^−/−^ for 20 min on ice surface, permeabilized with 0.1% Triton X-100/PBS^−/−^ for 5 min at 23 °C, then blocked in 2% BSA/PBS^−/−^ for 30 min at 23 °C. The fixed cells were sequentially incubated with appropriate Abs, (in 2% BSA/PBS, 1.5 h), washed 3 times with PBS^−/−^, and followed by incubation with either Cy-2 or rhodamine-conjugated secondary Abs (1:200) and DAPI (1:100) in 2% BSA/PBS^−/−^ for 1 h. Slides were analyzed and photographed by a fluorescence microscope (Olympus BX51, × 40). Background correction, and contrast adjustment of raw data images were performed using Photoshop (Adobe, CA, USA).

### Preparation of cellular extracts and Western blotting

Cells were grown to 70% confluence and serum starved (0.1% FCS, 16 h). After treatments, cell media was collected and floating cells where pelleted (8000 rpm, 1 min, 4 °C) and lysed in sample buffer 2X. In parallel, adherent cells were scraped into sample buffer 2X and combined with pelleted cells. The extracts were sonicated (50 W, 2 × 7 s), incubated on ice for 15 min, and boiled for 5 min. The samples were then subjected to 10% SDS-PAGE and Western blotting with the appropriate Abs. The blots were developed with HRP-conjugated anti-mouse or anti-rabbit Abs, using SuperSignal West Pico Chemiluminescent Substrate® from Thermo Scientific (Waltham, MA, USA) and then pictures of the blots taken using BioRad ChemiDoc MP System. Quantification of blots was done using ImageJ.

### Proliferation assays

To examine cell growth, melanoma cell lines (38 cell lines in Fig. 1) were seeded in six replicates in 96-well plates at 8,000 cells per well in 10% FCS medium. The next day, scrambled peptide or EPE peptide (final concentration of 10 μM) were added on the cells in 1% FCS medium. Fresh 1% FCS medium containing the same agents was replaced every day for four days and then cell proliferation was assessed using Cell-Titer-Glo reagent (Promega). The average number of EPE treated cells was divided by the average of Scrambled treated cells (%) as presented in Fig. 1.

### Cell viability assay

Cells were seeded at a density of 4000 cells per well into 96-well plates in complete medium. After 24 h, medium was replaced by 1% FCS containing appropriate treatments. Fresh medium containing the same agents was replaced every day. After 96 h, cell proliferation was assessed using the CellTiter-Glo reagent (Promega). IC_50_ values were determined using GraphPad Prism.

### Statistical Analyses

All statistical analyses were performed using Microsoft Excel to generate *P* values to determine significance (Student’s *t*-test).

### Data availability

No datasets were generated or analysed during the current study.

## Electronic supplementary material

supplementary information
