# Author Correction: Combined inhibition of MEK and nuclear ERK translocation has synergistic antitumor activity in melanoma cells

**DOI:** 10.1038/s41598-018-37931-7

**Published:** 2019-03-12

**Authors:** Rand Arafeh, Karen Flores, Alona Keren-Paz, Galia Maik-Rachline, Naomi Gutkind, Steven Rosenberg, Rony Seger, Yardena Samuels

**Affiliations:** 1 Weizmann Institute of Science, Rehovot, Israel; 2 National Cancer Institute, NIH, Bethesda, MD 20892 USA

Correction to: *Scientific Reports* 10.1038/s41598-017-16558-0, published online 27 November 2017

The Acknowledgements section in this Article is incomplete.

“Y.S. is supported by the Israel Foundation grant numbers 711631, the ERC (StG-335377), the MRA and by the Knell Family and the Hamburger Family. This work was supported by ISF and BSF to RS. R.S. is an incumbent of the Yale S. Lewine and Ella Miller Lewine Professional Chair for Cancer Research. R.A. is supported by the Clore Foundation”.

should read:

“Y.S. is supported by the Israel Foundation grant numbers 711631, the ERC (StG-335377), The Minerva Foundation Grant, the MRA and by the Knell Family and the Hamburger Family. This work was supported by ISF and BSF to RS. R.S. is an incumbent of the Yale S. Lewine and Ella Miller Lewine Professional Chair for Cancer Research. R.A. is supported by the Clore Foundation”.

