# Supplementary material for: Combined inhibition of MEK and nuclear ERK translocation has synergistic antitumor activity in melanoma cells
Source: Sci Rep. 2017 Nov 27;7:16345. doi: 10.1038/s41598-017-16558-0 (PMC5704016; doi:10.1038/s41598-017-16558-0)
Supplement: Supplementary file 1 — supplementary information [file 41598_2017_16558_MOESM1_ESM.doc]

**Supplementary Figures**

**Combined inhibition of MEK and nuclear ERK translocation has synergistic antitumor activity in melanoma cells**

Rand Arafeh^1#^, Karen Flores^1#^, Alona Keren-Paz^1^, Galia Maik-Rachline^1^, Naomi Gutkind^1^, Steven Rosenberg^2^, Rony Seger^1^ and Yardena Samuels^1^*

^1^ Weizmann Institute of Science, Rehovot, Israel

^2^ National Cancer Institute, NIH, Bethesda, MD 20892, USA

^#^ These authors contributed equally to this work

*To whom correspondence should be addressed: yardena.samuels@weizmann.ac.il

**Supplementary Table 1. *BRAF*, *NRAS* and *NF1* mutations in different melanomas cell lines.** WT: Wild-Type, N/A: Not Available


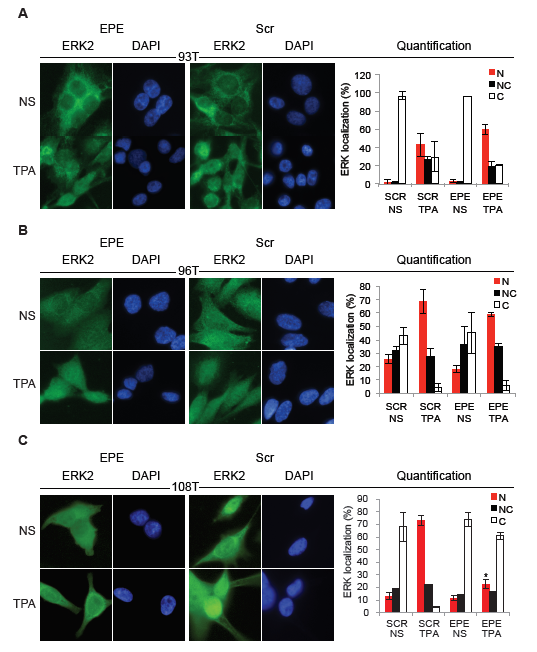


**Supplementary Figure 1. The effect of the EPE peptide on the nuclear translocation of *NF1* mutant and triple WT melanomas. (A) (B) and (C)** 93T, 96T (triple WT) and 108T (*NF1* mutant) cell lines were serum starved (14h), pretreated with EPE or Scr peptide (10 µM, 2 h), and stimulated with TPA (100 nM, 15 min) or left untreated (NS). Cells were then fixed and stained with αERK2 Abs and DAPI. Bars represent the average percentage of cells with mostly nuclear (N, red), nuclear and cytosolic (NC, black) or mostly cytosolic (C, white) staining. Error bars represent standard error of 2 independent experiments, **p* < 0.01. Quantification was done by counting at least three fields with > 150 cells

**Supplementary Figure 2. Synergistic effect of trametinib and EPE peptide combination in reducing the viability of melanoma cell lines.** Synergy between combined treatment of trametinib and EPE peptide in patient-derived melanoma cells. Dose response curves showing growth of melanoma cells treated with combination of trametinib and EPE peptide 10 µM (red), compared to trametinib treatment alone (black). Dots represent triplicates for every concentration point.
